# Sustained Obesity and Depressive Symptoms over 6 Years: Race by Gender Differences in the Health and Retirement Study

**DOI:** 10.3389/fnagi.2016.00312

**Published:** 2017-01-04

**Authors:** Julia D. Carter, Shervin Assari

**Affiliations:** ^1^Department of Epidemiology, University of Pittsburgh Graduate School of Public HealthPittsburgh, PA, USA; ^2^Department of Psychiatry, University of MichiganAnn Arbor, MI, USA; ^3^Center for Research on Ethnicity, Culture and Health, School of Public Health, University of MichiganAnn Arbor, MI, USA

**Keywords:** ethnic groups, Blacks, Whites, depressive symptoms, obesity, physical activity, self-rated health, well-being

## Abstract

**Background:** Although obesity and physical activity influence psychosocial well-being, these effects may vary based on race, gender, and their intersection. Using 6-year follow-up data of a nationally representative sample of adults over age of 50 in the United States, this study aimed to explore race by gender differences in additive effects of sustained high body mass index (BMI) and physical activity on sustained depressive symptoms (CES-D) and self-rated health (SRH).

**Methods:** Data came from waves 7, 8, and 10 (2004–2010) of the Health and Retirement Study (HRS), an ongoing national cohort started in 1992. The study enrolled a representative sample of Americans (*n* = 19,280) over the age of 50. Latent factors were used to calculate sustained high BMI and physical activity (predictors) and sustained poor SRH and high depressive symptoms (outcomes) based on measurements in 2004, 2006, and 2010. Age, education, and income were confounders. Multi-group structural equation modeling (SEM) was used to test the additive effects of BMI and physical activity on depressive symptoms and SRH, where the groups were defined based on race by gender.

**Results:** Group differences were apparent in the direction and significance of the association between sustained high BMI and depressive symptoms. The association between sustained high BMI and depressive symptoms was positive and significant for White women (*B* = 0.03, *p* = 0.007) and non-significant for White men (*B* = −0.03, *p* = 0.062), Black men (*B* = −0.02, *p* = 0.564) and Black women (*B* = 0.03, *p* = 0.110). No group differences were found in the paths from sustained physical activity to depressive symptoms, or from physical activity or BMI to SRH.

**Conclusion:** Sustained high BMI and high depressive symptoms after age 50 are positively associated only for White women. As the association between sustained health problems such as depression and obesity are not universal across race and gender groups, clinical and public health interventions and programs that simultaneously target multiple health problems may have differential effects across race by gender groups.

## Introduction

Increasing prevalence of obesity in the United States has become a significant public health concern (Office of Disease Prevention and Health Promotion, 2014). Although, research has consistently shown that obesity impacts psychological well-being (Wynne et al., 2014; Chang et al., 2016; Global BMIMC, 2016), a growing body of evidence suggests that psychosocial correlates of obesity may depend on race (Kodjebacheva et al., 2015; Murphy et al., 2015; Kelley et al., 2016), gender (Assari and Caldwell, 2015; Kodjebacheva et al., 2015), and their intersection (Assari and Lankarani, 2015a). Whether race by gender groups differ in the link between sustained obesity and psychosocial distress is still unknown.

Research has suggested that culture may alter the link between physical and emotional problems (Miyamoto et al., 2013; Park et al., 2013; Curhan et al., 2014; Kitayama et al., 2015). Compared to East Asians, negative emotions more strongly predict poor physical and mental health in the United States (Curhan et al., 2014). Even after controlling for sociodemographic variables, negative emotions elicit higher rates of inflammatory biomarkers in American adults than in Japanese counterparts (Miyamoto et al., 2013). It has been suggested that culture may moderate the association between expression of anger and biological health risk. For instance, Americans may have a higher biological health risk due to anger traits compared to the Japanese (Park et al., 2013; Kitayama et al., 2015). Similar differences have been found between Blacks and Whites (Johnson, 1989). In a series of studies, Assari has shown that depressive symptoms and negative affect better predict subsequent change in physical health outcomes such as chronic medical conditions and mortality for Whites than Blacks (Assari and Burgard, 2015; Assari et al., 2015b, 2016a,b; Assari and Lankarani, 2016b).

Despite a disproportionately higher level of stress, chronic disease, and low socioeconomic status associated with their minority status (Williams, 1999; Franks et al., 2006; Gold et al., 2006), Blacks seem to have a higher chance of thriving despite all their environmental adversities, as evidenced by better mental health (Teti et al., 2012; Ward et al., 2014). Continuous exposure to adversities may have resulted in a systematic resilience for Blacks that help them maintain psychological well-being, even in the presence of new difficulties. Stress has also shown weaker effects on depression among Black men, compared to White men, which is consistent with such resilience (Assari and Lankarani, 2016a).

Body dissatisfaction and perceived unattractiveness to others is a mechanism behind the comorbidity between high Body Mass Index (BMI) and negative emotions (Jackson et al., 2014; Webb et al., 2014a; Ehlinger and Blashill, 2016). Bandura's Social Cognitive Theory suggests that we are not what we are, or what we think we are, but what we think other people think about us (Bandura, 1986). The Social Ecological Framework also suggests that individuals' behaviors and emotions are shaped by their social interactions and environment (Ley et al., 2015). Although, high BMI influences body image perception and body dissatisfaction (Altintas et al., 2014; Coy et al., 2014; Das and Evans, 2014; Stephen and Perera, 2014; Webb et al., 2014a,b; Laus et al., 2015), there is a wealth of literature suggesting that these associations depend on gender (Altintas et al., 2014; Coy et al., 2014; Laus et al., 2015), race and ethnicity (Mikolajczyk et al., 2012; Richmond et al., 2012; Chithambo and Huey, 2013; Thomas et al., 2013; Fletcher, 2014; Gitau et al., 2014; Pope et al., 2014; Sabik, 2015; Blostein et al., 2016), and age (Altintas et al., 2014; Pope et al., 2014). Self-image and misperception of self also vary by race, gender (Nichols et al., 2009; Lynch and Kane, 2014; Baruth et al., 2015; Gustat et al., 2016), and culture (Capodilupo and Kim, 2014; Argyrides and Kkeli, 2015; Capodilupo, 2015; O'Neal et al., 2015). A wide range of social and cultural factors such as affirmations and expectations of social network, including but not limited to opposite sex (Capodilupo and Kim, 2014; O'Neal et al., 2015) and media (Capodilupo and Kim, 2014; Capodilupo, 2015), which differently shape thin body ideals across groups (Chithambo and Huey, 2013; Capodilupo and Kim, 2014; Argyrides and Kkeli, 2015), also have a role.

In an innovative approach introduced by Kendler and Gardner (2016) and then used by Assari et al. (2016c), latent factors can be used to model sustained health problems over time, and their correlates. Using structural equation modeling (SEM), in this study we investigated the links between sustained BMI, physical activity, depressive symptoms, and self-rated health over time, using multiple measures. In contrast to many studies in the literature that are typically cross-sectional or focused on the effect of baseline risk factor on subsequent trajectories, this statistical approach (Kendler and Gardner, 2016) allows for model associations between sustained health issues over time (please see our Methods section for more details about this approach and latent variables).

Despite the well-established links between multiple health problems such as BMI, SRH, physical activity, and depressive symptoms (Okosun et al., 2001; Stunkard et al., 2003; Jokela et al., 2016; Romo-Perez et al., 2016), little is known about race by gender differences in the links between sustained health problems over time. The current study aimed to test if sustained high depressive symptoms and poor SRH similarly reflect sustained high BMI and physical inactivity in Black men and women and White men and women, using a nationally representative cohort of U.S. adults over age of 50.

## Methods

### Design and setting

Data from the Health and Retirement Study (HRS), from the time period of 2004–2010, were used to study the effect of sustained BMI and physical activity on depressive symptoms and SRH (Assari et al., 2016c). The HRS is a longitudinal cohort study of a representative sample of American adults over the age of 50 that began in 1992. Further detailed information regarding the HRS can be found elsewhere (Heeringa and Connor, 1995).

### Ethics statement

The University of Michigan Institutional Review Board approved the HRS protocol for all years of data collection and all participants provided written informed consent for participation in the study.

### Participants and sampling

HRS participants were born between the years 1931 and 1941, inclusive. The HRS used a national area probability sample of United States households. The time period of 2004–2010 consisted of individuals in the HRS waves 7, 8, and 10. In the current study, we included individuals who considered their race to be White or Caucasian and Black or African American. At baseline (1992), there was a total sample size of 19,280 participants: 6705 White men (34.8%), 8860 White women (46.0%), 1468 Black men (7.6%), and 2247 Black women (11.7%).

### Data collection

Data were collected from participants using standard questionnaires and telephone or face-to-face interviews. Proxy interviews were used in situations in which participants were unable to respond for themselves. In addition to collecting extensive data on demographic, social, economic, and health information, the participants and their spouses were interviewed every two years.

### Measures

#### Body mass index

Body mass index was measured using participants' self-reported height (measured in feet and inches) and weight (measured in pounds). Height and weight were converted to meters and kilograms respectively, and BMI was calculated by dividing weight in kilograms by height in meters squared. The use of self-reported height and weight in the calculation of BMI has been validated (Stewart, 1982; Spencer et al., 2002).

#### Vigorous physical activity

Physical activity was measured by asking participants the following item regarding their participation in exercise: “On average over the last 12 months have you participated in vigorous activity or exercise three times a week or more? By vigorous physical activity, we mean things like sports, heavy housework, or a job that involves physical labor. (He and Baker, 2004; Jenkins et al., 2008)” Responses included 3 or more times a week, 1 or 2 times a week, 1 to 3 times a month, less than once a month, or never. Higher score reflects more sustained vigorous physical activity. A single-item self-reported physical activity measure has shown reliability and validity (Milton et al., 2011).

#### Depressive symptoms

Depressive symptoms were measured using the Center for Epidemiologic Studies Depression scale (CES-D). The CES-D scale is a self-report scale used to measure current level of depressive symptomatology (Radloff, 1977). A modified eight-item version of the CES-D scale was used for waves 7, 8, and 10 of the HRS; participants were given a CES-D score, with a higher score indicating more depressive symptomatology.

#### Self-rated health

Self-rated health was reported by asking respondents whether their health was excellent, very good, good, fair, or poor. SRH was dichotomized into two categories of poor/fair vs. good/very good/excellent (Stenholm et al., 2014). Research has demonstrated that there is a strong association between poor SRH and increased risk of mortality (Idler and Angel, 1990; Idler and Benyamini, 1997).

### Latent factors

#### Sustained health problems

For each health variable (BMI, SRH, physical activity, and depressive symptoms), we created a latent variable indicating vulnerability or a sustained health problem, using three observations in 2004, 2006, and 2010 as measured indicators for a latent variable. To estimate stability of each variable, the loadings were tested across time points. The latent variables were conceptualized as indicators for *stably high* level of health problem(s) over the follow up period. That is, higher scores of our latent variable were indicative of more sustained health problems over 6 years, which can be potentially seen as long-term vulnerability. For instance, the path from BMI to depressive symptoms reflects the effect of sustained high BMI on sustained high depressive symptoms over a 6-year period.

### Statistical note

Univariate and bivariate analyses were done in SPSS 20.0 (IBM Inc., Armonk, NY). Bivariate associations were tested using Pearson's correlation and paired samples *t*-test. We used AMOS 18.0 for multivariable analysis (Alessi, 2002; Arbuckle, 2009).

Structural equation modeling (SEM) was used for multivariable data analysis (Kline, 2011). In the first step, we fitted model with no constraining of paths across the groups. In the next step, we released constraints and compared the fit with that of the previous model. We also tested models where the error variances for corresponding pretest and posttest measures were correlated. As the fit did not improve in the second model, we reported the model with released constraints. In our models, we performed multi-group SEM analysis where the group was defined based on race by gender. We compared the path coefficients between the groups for statistically significant difference.

Independent variables included physical activity and BMI. The dependent variables of interest were CES-D and SRH. Covariates used in the model were age, education, and income. Latent factors were used for the independent and dependent variables by assessing the data at three cross-sectional time points (2004, 2006, 2010) during the study. Paths were drawn from the covariates to the dependent variables and from the independent variables to the dependent variables.

Fit statistics included were Chi square, the comparative fit index (CFI) [>0.90], the root mean squared error of approximation (RMSEA) [<0.06], and X2 to degrees of freedom ratio (Tabachnick and Fidell, 1996; Hu and Bentler, 1999; Lei and Lomax, 2005). Unstandardized and standardized regression coefficients were reported. We implemented full information maximum likelihood (FIML) to account for missing data. We considered *p* less than 0.05 as significant.

## Results

### Univariate analysis

Table 1 lists the descriptive statistics for all of the variables included in the study for the pooled sample as well as based on race and gender. As shown in the table, White men had consistently more sustained physical activity than White women and Black men and women. Additionally, Black women had the highest sustained BMI across race and gender.

**Table 1:** **Descriptive statistics in the pooled sample and based on race and gender**.

	***n***	**Mean**	**SD**	***n***	**Mean**	**SD**	***n***	**Mean**	**SD**	***n***	**Mean**	**SD**	***n***	**Mean**	**SD**
		**All**			**White Men**			**White Women**			**Black Men**			**Black Women**	
Age (2004)	19280	67.51	10.78	6705	67.58	10.25	8860	68.44	11.14	1468	64.87	10.13	2247	65.35	10.72
Education (1992)	19257	12.28	3.34	6694	12.80	3.30	8850	12.39	3.00	1467	10.88	4.16	2246	11.24	3.65
Income (2004)	19280	58932.35	101032.56	6705	72960.71	124145.63	8860	56116.10	94500.53	1468	48172.82	62464.07	2247	35205.97	50845.06
Activity 1	19261	1.92	1.30	6699	2.15	1.37	8852	1.82	1.26	1465	1.95	1.30	2245	1.57	1.10
Activity 2	17053	1.91	1.32	5922	2.16	1.40	7939	1.77	1.25	1218	2.05	1.35	1974	1.63	1.15
Activity 3	14008	1.96	1.31	4799	2.16	1.35	6526	1.84	1.26	1008	2.14	1.36	1675	1.73	1.21
BMI 1	18926	27.42	5.64	6676	27.56	4.69	8630	26.72	5.87	1453	27.68	5.11	2167	29.63	6.94
BMI 2	16818	27.83	5.83	5905	27.90	4.85	7772	27.24	6.10	1205	27.94	5.18	1936	29.90	7.18
BMI 3	13858	28.01	5.95	4790	28.13	5.01	6422	27.43	6.21	1001	28.12	5.61	1645	29.81	7.15
CES-D 1	17491	1.50	1.98	5904	1.15	1.73	8297	1.57	2.03	1222	1.68	2.01	2068	2.07	2.25
CES-D 2	15952	1.52	2.00	5460	1.17	1.75	7555	1.61	2.05	1074	1.67	1.92	1863	2.07	2.28
CES-D 3	12954	1.38	1.93	4375	1.09	1.71	6128	1.45	1.99	896	1.40	1.86	1555	1.90	2.16
SRH 1	19263	2.90	1.14	6701	2.82	1.14	8848	2.83	1.14	1468	3.16	1.12	2246	3.24	1.08
SRH 2	17050	2.90	1.13	5927	2.80	1.11	7932	2.84	1.13	1215	3.11	1.12	1976	3.28	1.08
SRH 3	14038	2.90	1.09	4811	2.84	1.08	6540	2.83	1.09	1010	3.06	1.04	1677	3.22	1.05

*Body Mass Index (BMI); Self-rated Health (SRH); Depressive Symptoms (CES-D)*.

### Bivariate analysis

Table 2 presents the correlation matrix of the study variables in the pooled sample. BMI and CES-D showed positive but weak correlation at all three time points with *p*<0.01 and *r* ranging from 0.072 to 0.098. BMI and SRH were also positively correlated at all time points with *p* < 0.01 and *r* ranging from 0.123 to 0.170.

**Table 2:** **Correlation matrix of the study variables in the pooled sample**.

		**1**	**2**	**3**	**4**	**5**	**6**	**7**	**8**	**9**	**10**	**11**	**12**	**13**	**14**	**15**
1	Age (2004)	1	−0.187^**^	−0.194^**^	−0.199^**^	−0.192^**^	−0.172^**^	−0.230^**^	−0.216^**^	−0.232^**^	0.022^**^	0.005	0.003	0.162^**^	0.164^**^	0.131^**^
2	Education (1992)		1	0.260^**^	0.188^**^	0.190^**^	0.163^**^	−0.059^**^	−0.052^**^	−0.060^**^	−0.226^**^	−0.227^**^	−0.211^**^	−0.322^**^	−0.318^**^	−0.280^**^
3	Income (2004)			1	0.142^**^	0.154^**^	0.141^**^	−0.017^*^	−0.021^**^	−0.022^**^	−0.134^**^	−0.128^**^	−0.129^**^	−0.196^**^	−0.199^**^	−0.176^**^
4	Activity 1				1	0.467^**^	0.393^**^	−0.076^**^	−0.086^**^	−0.100^**^	−0.188^**^	−0.172^**^	−0.154^**^	−0.312^**^	−0.276^**^	−0.238^**^
5	Activity 2					1	0.438^**^	−0.084^**^	−0.077^**^	−0.095^**^	−0.166^**^	−0.194^**^	−0.159^**^	−0.279^**^	−0.318^**^	−0.252^**^
6	Activity 3						1	−0.112^**^	−0.110^**^	−0.098^**^	−0.142^**^	−0.156^**^	−0.180^**^	−0.246^**^	−0.266^**^	−0.315^**^
7	BMI 1							1	0.892^**^	0.833^**^	0.077^**^	0.098^**^	0.097^**^	0.123^**^	0.146^**^	0.170^**^
8	BMI 2								1	0.859^**^	0.088^**^	0.094^**^	0.093^**^	0.143^**^	0.129^**^	0.154^**^
9	BMI 3									1	0.087^**^	0.095^**^	0.072^**^	0.157^**^	0.149^**^	0.123^**^
10	CES-D 1										1	0.589^**^	0.517^**^	0.423^**^	0.374^**^	0.329^**^
11	CES-D 2											1	0.557^**^	0.377^**^	0.424^**^	0.341^**^
12	CES-D 3												1	0.360^**^	0.368^**^	0.421^**^
13	SRH 1													1	0.686^**^	0.591^**^
14	SRH 2														1	0.634^**^
15	SRH 3															1

*BMI, Body Mass Index; SRH, Self-rated Health; CES-D, Depressive Symptoms*.

** *p < 0.01*,

* *p < 0.05*.

### Multivariable analysis

Our SEM showed good fit [*p* < 0.001, CMIN = 1606.146, DF = 256, CMIN/DF = 6.274, CFI = 0.988, RMSEA = 0.012 (90% CI = 0.011–0.012)]. Loadings for our independent and dependent variables are shown in Table 3. For sustained high BMI, loadings ranged from 0.76 to 1.00. Loadings ranged from 0.71 to 0.78 for sustained high depressive symptoms. The range of loadings for sustained poor SRH was 0.74 to 0.83. Loadings for sustained high physical inactivity ranged from 0.55 to 0.69.

**Table 3:** **Path coefficients (SEM) based on race and gender**.

			**White Men**		**White Women**		**Black Men**		**Black Women**	
			**B(SE)**	***P***	**B(SE)**	***P***	**B(SE)**	***P***	**B(SE)**	***P***
**PRIMARY PATHS**										
BMI-Sustained High Levels	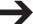	SRH-Sustained High Levels	0.04(0.00)	0.002	0.11(0.00)	<0.001	0.05(0.01)	0.042	0.12(0.00)	<0.001
BMI-Sustained High Levels	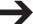	Depressive Symptoms-Sustained High Levels	−0.03(0.00)	0.062	0.03(0.00)	0.007	−0.02(0.01)	0.564	0.03(0.01)	0.110
**SECONDARY PATHS**										
Activity-Sustained High Levels	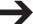	Depressive Symptoms-Sustained High Levels	−0.34(0.04)	<0.001	−0.32(0.04)	<0.001	−0.34(0.12)	<0.001	−0.26(0.10)	<0.001
Activity-Sustained High Levels	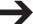	SRH-Sustained High Levels	−0.52(0.03)	<0.001	−0.44(0.03)	<0.001	−0.57(0.09)	<0.001	−0.43(0.06)	<0.001
**SES**										
Age	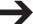	SRH Sustained High Levels	0.04(0.00)	0.004	0.09(0.00)	<0.001	−0.10(0.00)	0.007	0.05(0.00)	0.047
Age	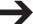	Depressive Symptoms -Sustained High Levels	−0.09(0.00)	<0.001	−0.05(0.00)	<0.001	−0.23(0.01)	<0.001	−0.16(0.00)	<0.001
Education	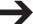	SRH Sustained High Levels	−0.24(0.00)	<0.001	−0.22(0.00)	<0.001	−0.16(0.01)	<0.001	−0.21(0.01)	<0.001
Education	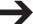	Depressive Symptoms-Sustained High Levels	−0.18(0.01)	<0.001	−0.20(0.01)	<0.001	−0.18(0.01)	<0.001	−0.22(0.01)	<0.001
Income	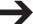	SRH Sustained High Levels	−0.04(0.00)	<0.001	−0.07(0.00)	<0.001	−0.14(0.00)	<0.001	−0.09(0.00)	<0.001
Income	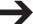	Depressive Symptoms-Sustained High Levels	−0.06(0.00)	<0.001	−0.05(0.00)	<0.001	−0.12(0.00)	<0.001	−0.12(0.00)	<0.001
**LOADINGS**										
BMI-Sustained High Levels	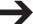	BMI 1	0.87		0.92		0.76		0.89	
BMI-Sustained High Levels	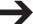	BMI 2	0.86(0.02)	<0.001	0.91(0.01)	<0.001	0.85(0.08)	<0.001	0.89(0.04)	<0.001
BMI-Sustained High Levels	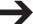	BMI 3	1.00(0.03)	<0.001	0.97(0.02)	<0.001	1.00(0.12)	<0.001	0.96(0.05)	<0.001
Depressive Symptoms-Sustained High Levels	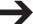	Depressive Symptoms 1	0.77		0.74		0.71		0.75	
Depressive Symptoms-Sustained High Levels	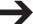	Depressive Symptoms 2	0.77(0.03)	<0.001	0.75(0.02)	<0.001	0.77(0.07)	<0.001	0.78(0.05)	<0.001
Depressive Symptoms-Sustained High Levels	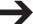	Depressive Symptoms 3	0.77(0.03)	<0.001	0.75(0.02)	<0.001	0.75(0.07)	<0.001	0.75(0.05)	<0.001
SRH-Sustained High Levels	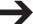	SRH 1	0.81		0.83		0.77		0.74	
SRH-Sustained High Levels	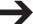	SRH 2	0.82(0.02)	<0.001	0.83(0.01)	<0.001	0.76(0.05)	<0.001	0.80(0.04)	<0.001
SRH-Sustained High Levels	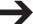	SRH 3	0.80(0.02)	<0.001	0.81(0.02)	<0.001	0.77(0.05)	<0.001	0.77(0.04)	<0.001
Activity-Sustained High Levels	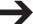	Activity 1	0.62		0.65		0.55		0.59	
Activity-Sustained High Levels	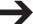	Activity 2	0.66(0.04)	<0.001	0.67(0.03)	<0.001	0.62(0.09)	<0.001	0.62(0.07)	<0.001
Activity-Sustained High Levels	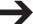	Activity 3	0.69(0.04)	<0.001	0.67(0.03)	<0.001	0.60(0.10)	<0.001	0.60(0.08)	<0.001

*Body Mass Index (BMI); Self-rated Health (SRH)*.

Table 3 also displays the two primary paths of interest in the current study; (1) the association between sustained high level of BMI and high depressive symptoms, and (2) the association between sustained high level of BMI and sustained poor SRH. There were group differences in the association between sustained BMI and CES-D. The association was significant and positive for White women (*B* = 0.03, *p* = 0.007), negative and non-significant for White men (*B* = −0.03, *p* = 0.062), negative and non-significant among Black men (*B* = −0.02, *p* = 0.564) and positive and non-significant among Black women (*B* = 0.03, *p* = 0.110). The association between sustained BMI and SRH were universal with no considerable group differences.

Table 3 also displays the secondary paths of interest, which include the association between sustained physical activity and CES-D and the association between sustained physical activity and SRH. These paths were systematic and demonstrated no group differences (all *p* < 0.0001).

As presented in Table 3, the three covariates included in the SEM were age, education, and income. For Black men, age was protective for SRH (*B* = −0.10, *p* = 0.007). For all other groups, age was a risk factor, with B ranging from 0.04 to 0.09. The effect of age on CES-D was systematic (*p* < 0.0001, B ranging from −0.23 to −0.05) and showed no group differences. Income and education were consistently protective for CES-D and SRH.

**Figure 1:**
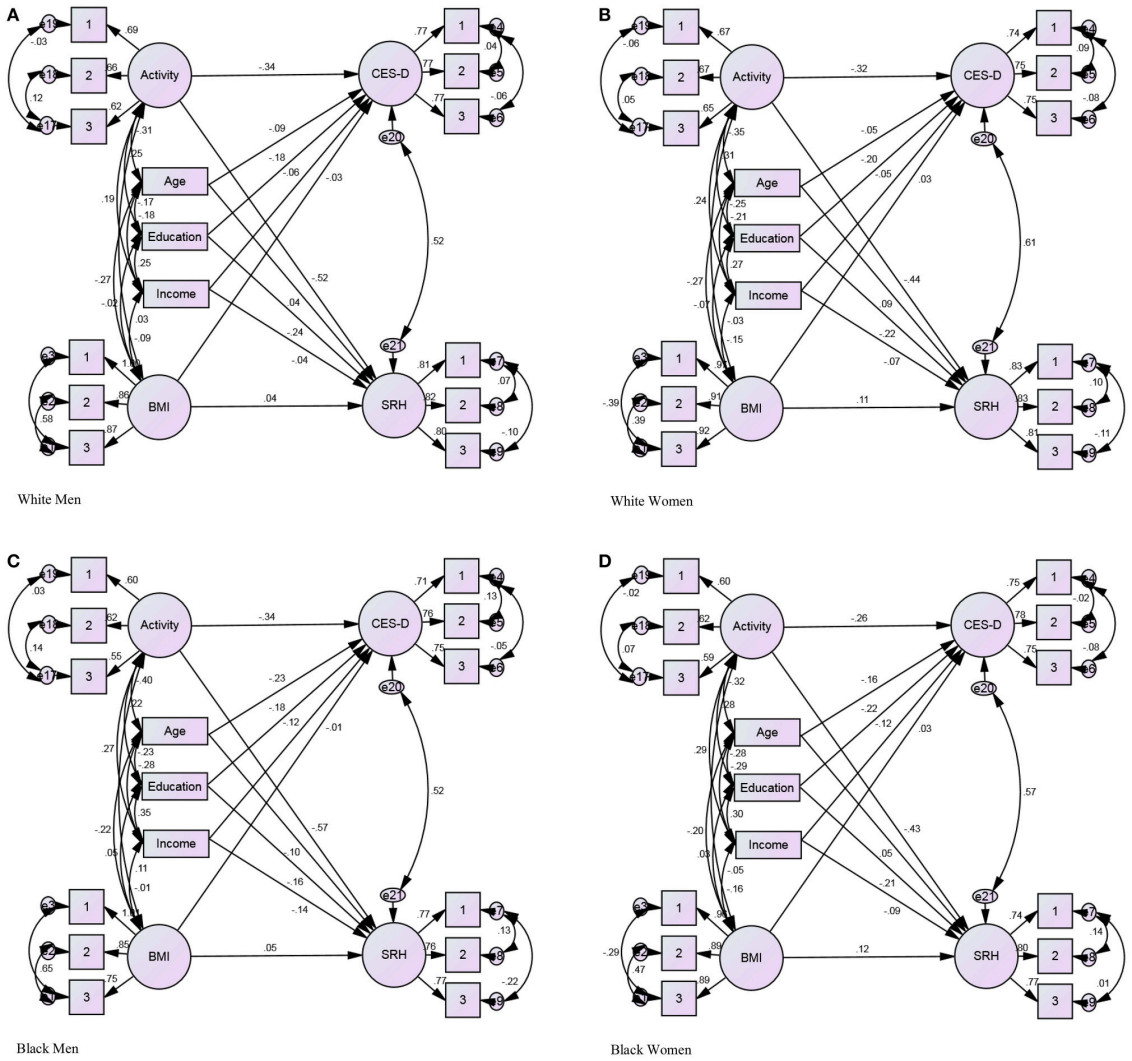
**Structural Equation Model (SEM) based on race by gender**. Dependent variables: Activity, and BMI. Independent variables: CES-D, and SRH. Covariates: Age, Education, and Income. *P* < 0.001, CMIN = 1606.146, DF = 256, CMIN/DF = 6.274, CFI 0.988, RMSEA 012 (011–012). **(A)** White Men. **(B)** White Women. **(C)** Black Men **(D)** Black Women.

Figures 1A–D illustrate the Structural Equation Model (SEM) for race by gender group. For example, in each of the four figures, the primary paths of interest are exhibited using arrows from sustained BMI to CES-D and sustained high BMI to sustained poor SRH. The secondary paths of interest were from sustained physical inactivity to sustained high depressive symptoms and sustained physical activity to sustained poor SRH. The path from BMI to CES-D was positive and significant for White women, marginal and negative for White men, and nonsignificant for Black women (positive) and Black men (negative).

## Discussion

The current study used a nationally representative sample of Americans older than 50 to assess whether sustained high levels of depressive symptoms and poor SRH universally reflect sustained high BMI and physical inactivity across race by gender groups. The results suggest that sustained high BMI had a positive and significant association with sustained high level of depressive symptoms in White women but not in White men, Black men, and Black women. The effect of sustained physical activity on sustained high level of depressive symptoms and SRH, as well as the effect of sustained BMI on sustained poor SRH, were, however, universal and similar across race by gender groups.

These findings suggest that demographic factors, environment, or cultural characteristics may influence how sustained obesity and depressive symptoms are associated (Park et al., 2013). Previous cross-sectional (Gavin et al., 2010; Hicken et al., 2013; Assari, 2014a,c; Assari and Caldwell, 2015; Kodjebacheva et al., 2015) and longitudinal (Hawkins et al., 2015) research had shown that the link between obesity and depressive symptoms depends on the intersection of race and gender (Assari, 2014b). The way in which physical activity shapes our perceived health is, however, universal. If an individual is active, he or she will feel healthy and less depressed, irrespective of group membership. Similarly, an individual with high BMI will feel less healthy regardless of group. For sustained obesity and depressive symptoms, comorbidity is not universal.

These findings coincide with the results of other studies on the differential effects of the association between obesity and major depressive disorder by race and gender (Gavin et al., 2010; Hicken et al., 2013; Assari, 2014a,b,c; Assari and Caldwell, 2015; Hawkins et al., 2015; Kodjebacheva et al., 2015). The results of this study in Black men and women are in agreement with a number of studies examining the “Jolly Fat” hypothesis, which supports that higher body mass index and obesity in women is associated with less depression (Jasienska et al., 2005; Kim et al., 2010; Yu et al., 2011). This hypothesis is also demonstrated among adolescent girls (Revah-Levy et al., 2011) as well as adult and elderly populations in Asian countries (Li et al., 2004; Kim et al., 2010; Yu et al., 2011). The “Jolly Fat” hypothesis was also reflected among aging Asian men (Li et al., 2004; Han et al., 2009; Dong et al., 2013). One study found that the desire to be of smaller size was not as great for Blacks compared to Whites, and Black women tended to feel that their size was considered satisfactory by their significant others (Kemper et al., 1994). Black women have more positive attitudes toward obesity and less internalized stigmatization (Latner et al., 2005).

Culture may influence cognitive and emotional elements that are essential for the perception of obesity and associated weight management behaviors (Assari and Lankarani, 2015a,b). One study found that urban, obese Black men who felt healthy or had fewer comorbid conditions had a greater misperception of healthy weight (Godino et al., 2010). Another study reported that about half of young African-American men with normal BMI desired to be heavier, while approximately 60% of overweight men were satisfied or wished to be heavier (Gilliard et al., 2007). The absence of strong negative social pressure combined with a positive body image perception among Black women (Kumanyika et al., 1993) and the desire of Black men to be of larger size (Jackson et al., 2010) contribute to a sustained higher BMI in this population. In addition, James Jackson has hypothesized that compared to Whites, Blacks engaged in unhealthy behaviors to cope with stressors of living in chronically stressful environments (Jackson et al., 2010; Mezuk et al., 2013). Over the life course, Black men demonstrate increased rates of smoking, alcohol consumption, and drug use, while Black women respond by overeating (Jackson et al., 2010). However, in the current study, there were no gender by race differences.

Much of the literature concerning the effects of life's adversities on Blacks document the disadvantages and weaknesses but hardly the strengths that have resulted in this population. Examples include forms of “double jeopardy” for Blacks in health and healthcare (Kirby and Kaneda, 2013) and academic learning (Taylor and Walton, 2011), the adverse effects of racism and discrimination (Williams, 1999; Chae et al., 2011; Gibbons et al., 2012), and the cumulative negative effects of multiple disadvantages (Zemore et al., 2011; Pais, 2014; Umberson et al., 2014). However, Teti et al. found that Black men who tackled challenges such as racism, incarceration, and unemployment demonstrated resilience amid these stressors (Teti et al., 2012). It has also been found that Black women exhibited resilience in spite of traumatic experiences (Henderson et al., 2015) and in order to cope with depression (Ward et al., 2014). It is this resilience among Blacks that allows this population to thrive and remain optimistic, thus resulting in better mental health outcomes than their counterparts. However, the resilience that this population demonstrates is not specific to the measures in the current study, but rather a resilience that is displayed across a variety of risk factors and outcomes. It is a systemic resilience as a result of the life course, and it reflects the contextual factors of one's life. James Jackson developed the Law of Small Effects, an explanation that suggests that physical and mental health disparities are effects of accumulated small differences through the life course as Blacks age (Jackson, 2011; Brown et al., 2014). Because the Law of Small Effects indicates that there is no single cause of health disparities but rather an accumulation of a variety of factors (Jackson, 2011; Brown et al., 2014), we propose that interventions will influence Blacks with smaller effects than Whites, thus the Law of Small Effects.

Corey Keyes hypothesizes a “Black advantage” in mental health, possibly due to flourishing in the presence of adversity (Keyes, 2007), to explain Blacks' lower rates of common mental disorders and a greater mental resilience despite adversities, stress, discrimination, and other risk factors (Keyes, 2009). Across all age cohorts, family satisfaction and contact with friends were found to be the most important contributing factors of general life satisfaction for Blacks (Adams and Jackson, 2000). Black-White differences may be due to culture, which shapes resilience (Keyes, 2009; Teti et al., 2012; Ward et al., 2014; Henderson et al., 2015), body image and perception (Altintas et al., 2014; Coy et al., 2014; Das and Evans, 2014; Stephen and Perera, 2014; Webb et al., 2014b; Laus et al., 2015), and social support (Adams and Jackson, 2000), all influencing mental health. Culture is a powerful influence on health outcomes as described by Kitayama et al. in the cultural moderation hypothesis (Park et al., 2013).

There are public health implications for the results of this study (Leon et al., 2014; Assari et al., 2015a; Krishna et al., 2015). Although, Black men and women with sustained high BMI do not report high depressive symptoms, there should still exist efforts to reduce the sustained high levels of BMI among Black men and women, similar to Whites. In order to reduce burden of obesity, we need multidisciplinary approaches that address the context, culture, and environment of populations that may be allowed higher body mass without stigma. Active involvement, partnership and communication with Black communities is vital to better understand cultural factors that may operate as barriers for obesity prevention in subpopulations.

Clinical and public health interventions that target healthy BMI may have differential effects on comorbid health outcomes for Blacks compared to Whites. Tailoring according to group membership may influence the association between high BMI and mental health needs. Therefore, universal interventions may not be ideal for diverse populations, as Whites and Blacks with high BMI have different patterns of comorbidities. To maximize benefits, interventions and programs may be tailored to race and gender to match that of the target population. One study that implemented a weight loss intervention among Black women reported that those with the greatest fat mass loss improved insulin sensitivity while those with fat mass gain, which was common, had reduced insulin sensitivity following the 6-month program (Leon et al., 2014). These results demonstrate that additional support may be beneficial for Black women in weight loss programs who fail to achieve optimal weight loss goals.

This study is subject to a few limitations. Attrition is a concern in longitudinal cohort studies. Loss to follow-up may have led to missing data that may skew the results. Another limitation is the use of self-reported weight and height for BMI calculation. Although, research finds that this measure is valid, self-reported data is still subject to bias and under/overestimation. Also, the use of the single-item physical activity measure may fail to capture participants' true physical activity levels. Lastly, the unbalanced sample size resulted in different statistical power across groups. The current study makes a significant contribution to the existing literature as it is one of the first studies on sustained health risk over time. A nationally representative sample, large sample size, length of follow up, and the intersectional approach are key strengths in the current study. This study used an innovative statistical approach introduced by Kendler (Kendler and Gardner, 2016). Further research should be done on risk factors and outcomes associated with sustained health issues over time.

In conclusion, how sustained high level of BMI and sustained depressive symptoms are associated varies across race by gender groups. Sustained high depressive symptoms better reflect sustained high BMI level for White women than White men, Black men, or Black women. The association between sustained BMI and depressed affect is not uniform, but specific to the race by gender intersection. Clinical and public health interventions and programs that are tailored to the target populations may be more effective.

## Author contributions

JC: Drafted the manuscript and revised the manuscript; SA: Developed the conceptual model of the study, analyzed the data, and contributed to the drafts. Both authors confirmed the final version of the manuscript.

## Funding

Assari is supported by the Heinz C. Prechter Bipolar Research Fund and the Richard Tam Foundation at the University of Michigan Depression Center.

### Conflict of interest statement

The authors declare that the research was conducted in the absence of any commercial or financial relationships that could be construed as a potential conflict of interest.
